# A Practical Guide to Immunoassay Method Validation

**DOI:** 10.3389/fneur.2015.00179

**Published:** 2015-08-19

**Authors:** Ulf Andreasson, Armand Perret-Liaudet, Linda J. C. van Waalwijk van Doorn, Kaj Blennow, Davide Chiasserini, Sebastiaan Engelborghs, Tormod Fladby, Sermin Genc, Niels Kruse, H. Bea Kuiperij, Luka Kulic, Piotr Lewczuk, Brit Mollenhauer, Barbara Mroczko, Lucilla Parnetti, Eugeen Vanmechelen, Marcel M. Verbeek, Bengt Winblad, Henrik Zetterberg, Marleen Koel-Simmelink, Charlotte E. Teunissen

**Affiliations:** ^1^Clinical Neurochemistry Laboratory, Institute of Neuroscience and Physiology, The Sahlgrenska Academy, University of Gothenburg, Mölndal, Sweden; ^2^Neurobiology Laboratory, Centre for Memory Resources and Research (CMRR), Groupement Hospitalier Est (GHE), Hôpitaux de Lyon, Université Lyon 1, CNRS UMR5292, INSERM U1028, Lyon, France; ^3^Department of Neurology, Donders Institute for Brain, Cognition and Behaviour, Radboud University Medical Center, Nijmegen, Netherlands; ^4^Department of Laboratory Medicine, Donders Institute for Brain, Cognition and Behaviour, Radboud Alzheimer Centre, Nijmegen, Netherlands; ^5^Laboratory of Clinical Neurochemistry, Department of Medicine, Section of Neurology, University of Perugia, Perugia, Italy; ^6^Reference Center for Biological Markers of Dementia (BIODEM), Institute Born-Bunge, University of Antwerp, Antwerp, Belgium; ^7^Department of Neurology and Memory Clinic, Hospital Network Antwerp (ZNA) Middelheim and Hoge Beuken, Antwerp, Belgium; ^8^University of Oslo, Oslo, Norway; ^9^Department of Neurology, Akershus University Hospital, Lørenskog, Norway; ^10^Department of Neuroscience, Institute of Health Science, Dokuz Eylul University, Izmir, Turkey; ^11^Department of Neuropathology, University Medical Center, Göttingen, Germany; ^12^Division of Psychiatry Research, University of Zurich, Schlieren, Switzerland; ^13^Department of Psychiatry and Psychotherapy, Universitätsklinikum Erlangen, Friedrich-Alexander Universität Erlangen-Nürnberg, Erlangen, Germany; ^14^Paracelsus-Elena Klinik, Kassel, Germany; ^15^Department of Neuropathology, University Medical Center Göttingen, Göttingen, Germany; ^16^Department of Neurodegeneration Diagnostics, Medical University of Białystok, Białystok, Poland; ^17^ADx NeuroSciences, Ghent, Belgium; ^18^Karolinska Institutet, Stockholm, Sweden; ^19^Institute of Neurology, University College London, London, UK; ^20^Neurochemistry Laboratory and Biobank, Department of Clinical Chemistry, Neurocampus Amsterdam, VU University Medical Center, Amsterdam, Netherlands

**Keywords:** immunoassays, method validation, precision, limits of quantitation, robustness

## Abstract

Biochemical markers have a central position in the diagnosis and management of patients in clinical medicine, and also in clinical research and drug development, also for brain disorders, such as Alzheimer’s disease. The enzyme-linked immunosorbent assay (ELISA) is frequently used for measurement of low-abundance biomarkers. However, the quality of ELISA methods varies, which may introduce both systematic and random errors. This urges the need for more rigorous control of assay performance, regardless of its use in a research setting, in clinical routine, or drug development. The aim of a method validation is to present objective evidence that a method fulfills the requirements for its intended use. Although much has been published on which parameters to investigate in a method validation, less is available on a detailed level on how to perform the corresponding experiments. To remedy this, standard operating procedures (SOPs) with step-by-step instructions for a number of different validation parameters is included in the present work together with a validation report template, which allow for a well-ordered presentation of the results. Even though the SOPs were developed with the intended use for immunochemical methods and to be used for multicenter evaluations, most of them are generic and can be used for other technologies as well.

## Introduction

Biochemical markers (biomarkers) play a central role in the decision-making in clinical medicine. Examples include making a clinical diagnosis, initiating and monitoring treatment, predicting prognosis or disease recurrence after treatment. Among brain disorders, the Alzheimer’s disease (AD) field is in the good situation that a panel of cerebrospinal fluid (CSF) biomarkers is at hand, including the 42 amino acid variant of β-amyloid (Aβ42), total tau (T-tau), and phosphorylated tau (P-tau), and have in numerous studies been shown to have high diagnostic accuracy for AD also in the early stage of the disease (1). Except for an increasing use in clinical routine, these biomarkers are also used in clinical trials, both as diagnostic and as theragnostic markers (2). Last, these CSF biomarkers are applied in clinical studies on disease pathogenesis, and many research reports present novel biomarker candidates. The vast majority of such fluid biomarkers are low-abundance proteins, for which antibody-based immunoassays, often in the enzyme-linked immunosorbent assay (ELISA) format, is needed to get enough analytical sensitivity. However, to get reliable and reproducible results, rigorous control of assay performance is essential, which also should be presented in a standardized format.

Validation of a method is the confirmation by examination and the provision of objective evidence that the particular requirements for a specific intended use are fulfilled (3). It is important as it defines whether it will produce reliable results in the context of its intended use. This last item is sometimes overlooked; the intended use of a method needs to be carefully specified before any time consuming and costly validation experiments are performed. This notion is generic to any method. However, this paper will now focus on the validation of methods used to determine analyte concentrations in biofluids. The intended use for such a method could be to use the outcome as a diagnostic marker and in this case some evidence should be in place showing that there is a disease-dependent change in the analyte concentration in a biological sample. Furthermore, the magnitude of the change should have an impact on the acceptable variability of the method, i.e., if the change is small the higher is the demand on the precision and on the analytical sensitivity and specificity.

Much has been published on the topic of method validation but a consensus protocol on how to perform the task is yet to be found. This could be partly due to the fact that different analytical technologies have different requirements on which validation parameters that need to be addressed or that local initiatives by national societies in the clinical chemistry field were not discussed and spread at international level (4). For example, carryover should be investigated in a chromatography-based method while it is not applicable in an ELISA. The aim of the present work was to present straightforward step-by-step standard operating procedures (SOPs) for the validation of methods in which an analyte is determined in a biofluid matrix; the SOPs have been developed with the intention that they should be possible to follow without any advanced prior training.

This work is the main deliverable of the sub-task “Development of assay qualification protocols” in the BIOMARKAPD project supported by the European Union initiative Joint Programme – Neurodegenerative Disease Research. The BIOMARKAPD project aims for standardization of biomarker measurements for AD and Parkinson’s disease (PD), including pre-analytical and analytical procedures, assay validation, and development of reference measurement procedures (RMP) and certified reference materials (CRM) for harmonization of results across assay formats and laboratories. The work flow in the present project consisted of writing draft SOPs for each parameter relevant to validation of a method for determination of an analyte concentration in a biofluid. Task members were then asked to review and revise the SOPs, whereafter they were evaluated in at least three multicenter studies. End-users commented on the draft SOPs, and, after an additional round of reviews, final, consensus SOPs were produced which form the core of the current report. All members of the task were invited to critically revise the manuscript.

## Full vs. Partial Validation

Standard operating procedures for 10 different validation parameters are presented. If a method is developed in-house, a full validation should be performed, meaning that all parameters should be investigated. As a consensus agreement in the group, it was decided that a partial validation of a commercial assay should include all parameters except for robustness, which should have been covered by the manufacturer during method development. Even more limited partial validations may be eligible under other circumstances. For example, if a validated *in vitro* diagnostic (IVD) method is transferred to another laboratory to be run on a different instrument by a different technician it might be sufficient to revalidate the precision and the limits of quantification since these variables are most sensitive to the changes, while more intrinsic properties for a method, e.g., dilution linearity and recovery, are not likely to be affected.

It is also advisable to have a dialog with the client/sponsor to agree to what extent the method should be validated. Unfortunately, the standard ISO 15189 (20), which is designed for clinical laboratories, does not provide much rigor by only stating that “The validations shall be as extensive as are necessary to meet the needs in the given application or field of application.”

## Validation Report

If a laboratory is, or plan to be, accredited to some international standard there is usually a high demand on documentation. For example, in order to comply with the standard ISO 15189 “The laboratory shall record the results obtained and the procedure used for the validation (20).” To facilitate this and at the same time allow for a well-ordered presentation of the results a validation report template can be found in Data Sheet S1 in Supplementary Material. The template has been adapted from a Swedish handbook on method validation (5), with the permission of the authors. Below an outline of the 10 validation parameters is given and a short definition of each are presented in Table 1. To aid in the extraction of information from measurement data the Data Sheet S2 in Supplementary Material can be used.

**Table 1:** **Short description of the validation parameters for which SOPs are presented**.

	Parameter	Definition	Reference
1	Robustness	The ability of a method to remain unaffected by small variations in method parameters	(6)
2	Precision	The closeness of agreement between independent test results obtained under stipulated conditions	(7)
3	Trueness	The closeness of agreement between the average value obtained from a large series of test results and an accepted reference value	(7)
4	Uncertainty	A parameter associated with the result of a measurement, that characterizes the dispersion of the values could reasonably be attributed to the measurand	(8)
5	Limits of quantification	Highest and lowest concentrations of analyte that have been demonstrated to be measurable with acceptable levels of precision and accuracy	(6)
6	Dilutional linearity	Dilutional linearity is performed to demonstrate that a sample with a spiked concentration above the ULOQ can be diluted to a concentration within the working range and still give a reliable result	(6)
7	Parallelism	Relative accuracy from recovery tests on the biological matrix or diluted matrix against the calibrators in a substitute matrix	(6)
8	Recovery	The recovery of an anlayte in an assay is the detector response obtained from an amount of the analyte added to and extracted from the biological matrix, compared to the detector response obtained for the true concentration of the analyte in the solvent	(9)
9	Selectivity	The ability of the bioanalytical method to measure and differentiate the analytes in the presence of components that may be expected to be present	(9)
10	Sample stability	The chemical stability of an analyte in a given matrix under specific conditions for given time intervals	(9)

### Robustness

Robustness or ruggedness is the ability of a method to remain unaffected by small variations in method parameters. If the instructions from the manufacturer of a commercially available assay does not contain any information indicative of a robustness assessment the manufacturer should be contacted and asked to provide this information since it is likely that such data is available given that the method development was sound. In case of an in-house method, the robustness should be investigated as a part of the method development and the results should be reflected in the assay protocol before other validation parameters are investigated. The reason for this is that a validation is linked to an assay protocol and changes in the latter might demand a new validation to be performed.

#### Procedure

1. Identify critical parameters in the procedure, e.g., incubation times and temperatures.
2. Perform the assay with systematic changes in these parameters, one at the time, using the same set samples at each occasion.
3. If the measured concentrations do not depend on the changes, adjust the protocol by adding appropriate intervals, e.g., 30 ± 3 min or 23 ± 5°C, to the critical parameters.
4. If the changes systematically alter the measured concentrations, lower the magnitude of the changes until no dependence is observed. Incorporate the results into the protocol.

Note: if many critical steps are identified the number of experiments can be reduced using dedicated software, e.g., MODDE (Umetrics) or published methods (10).

### Precision

Precision is defined as “The closeness of agreement between independent test results obtained under stipulated conditions” (7). There are three different types of precisions depending on the stipulated conditions and these are repeatability (r), intermediate precision (Rw), and reproducibility (R). Repeatability is the variability observed when as many factors as possible, e.g., laboratory, technician, days, instrument, reagent lot, are held constant and the time between the measurements is kept to a minimum as opposed to reproducibility conditions where all factors are varied and measurements are carried out over several days. For intermediate precision, all factors except laboratory are allowed to vary and for clarity the factors changed should be stated in the validation report. Repeatability is sometimes called within-run or within-day precision while intermediate precision is also known as between-run or between day repeatability.

Precision is difficult to quantify and it is therefore the inversely related imprecision that is commonly reported. As measures of the imprecision it is usual to report both the SD and coefficient of variation (%CV) for the different levels of the measurand investigated with the condition as a subscript, e.g., %CV_Rw_. Analysis of variance (ANOVA) is used in the estimation of the imprecision and to facilitate in the calculations an excel file (Data Sheet S3 in Supplementary Material) has been created using the formulas in ISO 5725-2 (11).

#### Procedure

1. Collect samples with known high and low concentrations of the measurand. Pool samples if necessary.
2. Make 25 aliquots of each sample and store at −80°C pending analysis.
3. At day 1–5 measure 5 replicates on each sample. Note: the days need not to be consecutive, only different.
4. Insert data, separate days on different rows, in the excel file Data Sheet S3 in Supplementary Material that calculates the mean value, SD, %CV for both the repeatability and intermediate precision.

Five samples with different levels have been suggested as a general rule to cover a wide measuring range (7). However, it can be argued that if the levels are chosen with care, for example, one above and one below the decision limit, two samples might be enough. In addition, it is not always possible to obtain samples covering a wide range, e.g., when levels in patients and controls do not differ much or when these levels are still to be defined. If large volumes of the samples are available, more aliquots than the ones needed for the precision measurements can be prepared for use as internal quality control samples when the method has been put in service.

Other experimental schemes than the one suggested under points 2–3 in the procedure are possible, e.g., 12 replicates on 1 day and 3 replicates on 4 different days, or as the Clinical and Laboratory Standards Institute recommends, 2 separate runs on 20 days (total 40 runs) (12). The latter scheme will allow for more different factors to be explored, which will give a better estimate of the variability. At the same time, it is very impractical and expensive if the method is, e.g., a commercial ELISA kit where the number of calibrator curves that can be constructed in each kit-package is usually very limited.

### Trueness

Trueness is defined as “The closeness of agreement between the average value obtained from a large series of test results and an accepted reference value” (7). Ideally, the reference value is derived directly from a CRM or from materials that can be traced to the CRM. The quantity in which the trueness is measured is called bias (b), which is the systematic difference between the test result and the accepted reference value.

#### Procedure

1. Given that there exists a CRM, calculate the bias (*b*_CRM_) using formula (1) where the measured mean value ($\bar{\text{X}}$) is calculated from five replicates and *x*_ref_ is the assigned reference value.
2. If there exists an external quality control (QC) program, but no CRM, the bias (*b*_QC_) is calculated as the mean value of the deviations from the assigned QC values using formula (2). Note: the bias might be concentration-dependent and therefore *b*_QC_ should preferably be calculated using a longitudinal QC sample.

Formulas

(1) $$\begin{array}{l} b_{\text{CRM}}=\bar{\text{X-}}\;X_{\text{ref}} \end{array}$$

(2) $$\begin{array}{l} b_{\text{QC}}=\frac{\sum_{\text{i=1}}^{\text{n}}\left(X_{\text{i}}-X_{\text{QCi}}\right)}{n} \end{array}$$

where n is the number of measurements, *x*_i_ is the value measured in the laboratory, and *x*_QCi_ is the value from the *i*th sample in the QC program.

Once the bias is determined, it can be used to compensate the measured concentration resulting in a method without systematic effects (8). If the bias is constant over the measurement interval the bias is simply subtracted from the measured value and if the bias is proportional to the measured concentration the correction is done by multiplication of a factor determined from bias evaluations at different concentrations. Alternatively, the calibrators can be assigned new values to compensate for the bias. The total bias is the sum of two components originating from the method and the laboratory, respectively. When a CRM is available, manufacturers are obliged to calibrate their method against materials traceable to the CRM and then the total bias should in principle be equal to the laboratory bias.

### Uncertainty

The intermediate precision provides information about the dispersion characteristics of the results within a laboratory with no regard to the true value of a measurand in a sample. Therefore, in the absence of a CRM, the measurements rather deliver relative concentrations as opposed to absolute ones that can be achieved if the calibrators were traceable to a CRM. However, if different methods can be used for quantifying the same analyte and if a universal cutoff value is warranted there is a need for a CRM that can be used by the kit manufacturers to calibrate their methods against, in order to minimize the bias. This will also enable calculating absolute concentrations but the uncertainty in the results must then include not only the uncertainty from the method but also the uncertainty of the assigned value for the CRM.

#### Procedure

1. Given that there exists a CRM, calculate the combined uncertainty (*u*_c_) from the standard uncertainty of the precision (*u*_precision_) and the CRM (*u*_CRM_) using formula (3). Note: both *u*_precision_ and *u*_CRM_ have to be SDs. Note: this way of calculating the *u*_c_ assumes that the bias has been adjusted for as outlined in the trueness section above. Note: the results from the precision measurements can be used as an estimate of the uncertainty, e.g., *u*_precision_ = *s*_RW_.
2. Calculate the expanded uncertainty (*U*) using formula (4). Note: the coverage factor (*k*) is set to 2 for a confidence interval of approximately 95%. Note: the coverage factor for a given confidence interval is dependent on the degrees of freedom. Details on this and coverage factors for other confidence intervals can be found elsewhere (8).

Formulas

(3) $$\begin{array}{l} u_{\text{c}}=\sqrt{{\left(u_{\text{precision}}\right)}^{2}+{\left(u_{\text{CRM}}\right)}^{2}} \end{array}$$

(4) $$\begin{array}{l} U=k\cdot u_{\text{c}} \end{array}$$

### Limits of quantification

The working range for a method is defined by the lower and upper limits of quantification (LLOQ and ULOQ, respectively). At least for the LLOQ, there is more than one definition and these can be classified as either determined based on the signals from the instrument or the calculated concentrations from samples. For the former, a number of blank samples are analyzed and the average and SD of the signal are calculated (13).

#### Procedure

1. Run 16 blank samples (immunodepleted matrix or sample diluent)
2. Calculate the mean and SD of the signal.
3. Determine the concentration based on a signal of 10 SDs above the mean of the blank. Note: this procedure gives only the LLOQ but not the ULOQ.

To determine the concentration based on a signal the inverse of the calibration function must be used. The two most common models used in immunochemical calibrations are the four and five parametric logistic models. The four parametric function and its inverse are:

(5) $$\begin{array}{ll} \text{Signal} & =\frac{A-D}{1+{\left(\frac{\text{Concentration}}{C}\right)}^{\text{B}}}+D\Leftrightarrow \text{Concentration}=C{\left(\frac{A-D}{\text{Signal}-D}-1\right)}^{\frac{\text{1}}{\text{B}}} \end{array}$$

For the five-parameter logistic model the corresponding functions are:

(6) $$\begin{array}{l} \text{Signal}=\frac{A-D}{{\left(1+{\left(\frac{\text{Concentration}}{C}\right)}^{\text{B}}\right)}^{\text{E}}}+D\Leftrightarrow \text{Concentration}\;=C{\left({\left(\frac{A-D}{\text{Signal}-D}-1\right)}^{\frac{\text{1}}{\text{E}}}-1\right)}^{\frac{\text{1}}{\text{B}}} \end{array}$$

The parameters *A*–*E* should be available from the software used for data acquisition and analysis.

Based on the concentrations the LLOQ and ULOQ can be defined as the endpoints of an interval in which the %CV is under a specific level with the option of a higher %CV at the endpoints (9, 14).

#### Procedure

1. Analyze, in duplicates, samples with very low and very high concentrations of the measurand.
2. Calculate the average concentration and %CVs for the samples.
3. Make a scatter plot of the %CV as a function of concentration for all samples.
4. Determine the LLOQ by identifying the lowest mean level above which the %CV < 20% for the greater majority of the samples.
5. Determine the ULOQ by identifying the highest mean level below which the %CV < 20% for the greater majority of the samples.

### Dilution linearity

Dilution linearity is performed to demonstrate that a sample with a spiked concentration above the ULOQ can be diluted to a concentration within the working range and still give a reliable result. In other words, it determines to which extent the dose–response of the analyte is linear in a particular diluent within the range of the standard curve. Thereby dilution of samples should not affect the accuracy and precision. At the same time, the presence of a hook effect, i.e., suppression of signal at concentrations above the ULOQ, is investigated.

#### Procedure

1. Spike three samples (undiluted) with calibrator stock solution, as high as possible. Note: if possible, spike (undiluted) samples with 100- to 1000-fold the concentration at ULOQ using the calibrator stock solution. Biological samples can also be diluted less than the prescribed concentration, if an assay allows to.
2. Make serial dilutions of the spiked samples, using sample diluent in small vials until the theoretical concentration is below LLOQ. Note: the dilution should be performed using vials and not directly in the wells of the ELISA plate.
3. Analyze the serial dilutions in duplicates and compensate for the dilution factor.
4. Calculate for each sample the mean concentration for the dilutions that fall into the range of LLOQ and ULOQ. Moreover, calculate for each sample the %Recovery for the calculated concentration at each dilution. Note: the calculated concentration for a dilution that fall into the range of LLOQ and ULOQ should be within the acceptance criteria for the precision defined in the “SOP for fit-for-purpose” as should the calculated SD. Also, plot the signal against the dilution factor to investigate if the signal is suppressed at much higher concentrations than the ULOQ of the measurand (“hook effect”).

Dilution linearity should not be confused with linearity of quantitative measurement procedures as defined by CLSI (15), which concerns the linearity of the calibration curve.

### Parallelism

Conceptually parallelism and dilution linearity are similar. The major difference is that in the dilution linearity experiments the samples are spiked with the analyte to such a high concentration that after dilution the effect of the sample matrix is likely to be negligible. For parallelism, on the other hand, no spiking is allowed but only samples with high endogenous concentrations of the analyte must be used. However, the concentrations must be lower than the ULOQ. The goal of investigating the parallelism is to ascertain that the binding characteristic of the endogenous analyte to the antibodies is the same as for the calibrator.

#### Procedure

1. Identify four samples with high, but below ULOQ, endogenous concentration of the measurand.
2. Make at least three, two-fold serial dilutions using sample diluent in reaction vials until the calculated concentration is below LLOQ. Note: the dilution factor should be adjusted to obtain concentrations that are evenly spread over the standard curve. For example, dilution factor 10 if a standard curve includes values between 0.1 and 200 pg/ml.
3. Analyze the neat samples and the serial dilutions in duplicates, in the same run, and compensate for the dilution factor.
4. For each sample, calculate the %CV using results from neat sample and the dilutions.

There are different views on what the acceptance criteria for the %CV should be for showing the presence of parallelism. It has been suggested that %CV ≤ 30% for the samples in the dilution series is enough (14, 16) while others advocate a lower level of below 20% (17) or within the range 75–125% compared to the neat sample (18). None of these suggestions, however, relate the acceptance criteria to the precision of the method under investigation.

### Recovery

The recovery of an analyte in an assay is the detector response obtained from an amount of the analyte added to and extracted from the biological matrix, compared to the detector response obtained for the true concentration of the analyte in solvent (9). A spike recovery test is conducted to investigate if the concentration–response relationship is similar in the calibration curve and the samples. A bad outcome of the test suggests that there are differences between the sample matrix and calibrator diluent that affects the response in signal. Data obtained from this study could help to find a diluent mimicking the biological sample in which the calibrator and the native protein give the comparable detector signals all along the measuring range.

#### Procedure

1. Collect five samples were the concentrations of the measurand have previously been determined and divide each sample into 4 aliquots.
2. Spike three of the aliquots, using calibrator stock solution, to expected concentrations that are evenly distributed over the linear range of the standard curve (low, medium, high). Note: all additions should be in the same volume, preferable <10% of the sample volume. The same volume of measurand-free calibrator diluent must also be added to the neat sample (fourth aliquot) to compensate for the dilution. Note: the theoretical concentration in the spiked samples should be lower than the ULOQ. Different spiking concentrations should be used to investigate possible dependency on the amount of added substance. The low spike should be slightly higher than the lowest reliable detectable concentration. Note: alternatively, samples can be spiked after dilution if there is limited availability of the calibrator and high working dilutions.
3. Analyze both the neat and spiked samples in the same run. Dilute each sample as advised for each assay to be used,
4. Calculate the recovery using formula (7). Note: acceptance range for the recovery is usually 80–120%.

(7) $$\%\text{ Recovery}=\frac{\begin{array}{c} \text{Measured concentratio}{\text{n}}_{\text{spiked sample}}-\text{Measuered concentratio}{\text{n}}_{\text{neat sample}} \end{array}}{\text{Theoretical concentratio}{\text{n}}_{\text{spiked}}}\times 100$$

### Selectivity

Selectivity can be defined as “the ability of the bioanalytical method to measure and differentiate the analytes in the presence of components that may be expected to be present” (9). The terms “selectivity” and “specificity” are often used interchangeably while their significances are different. Selectivity is something that can be graded while specificity is an absolute characteristic. Specificity can be considered as the ultimate selectivity. For this reasons, selectivity should be preferred and is the recommended terminology. Of the different validation parameters the selectivity is in principle the only one for which a certain amount of knowledge about the analyte and related substances is demanded. For example, if the analyte is a peptide of a specific length do slightly longer or shorter peptides also give rise to a signal in the assay? Do metabolites of the analyte or post translational modifications of a protein analyte interfere with the assay?

#### Procedure

1. Identify substances that are physiochemically similar to the one that the assay is developed for.
2. Investigate to what degree the measurements are interfered by spiking samples with substances identified in step 1. Note: if information is available regarding the endogenous concentration of an investigated substance the spiking concentration should be at least two times the reference limit. Otherwise a titration is recommended.
3. For antibody-based methods, an epitope mapping should be performed.

### Sample stability

Sample handling prior to analysis has the potential to dramatically influence the results of a measurement. For this reason, it is important to investigate if different storage conditions contribute to systematic errors in order to provide the clinicians with adequate sample collection and transport instructions. The information gathered will also be useful once the sample reaches the laboratory, i.e., how it should be stored until analysis or pending a possible need for a re-run. Examples of factors that potentially affect the results of an analysis, but are not included in the following procedure includes, sample tube, type of plasma anticoagulant, gradient effects (concerns CSF samples), centrifugation conditions, extended mixing, and diurnal variations. If data are not available on how these factors influence the measurement the sample instructions should be written in a way to prevent variations potentially induced by these.

#### Procedure

1. Repeat the following steps for three independent samples, preferably with different concentrations of the measurand (low, medium, high).
2. Divide the sample into nineteen aliquots with equal sample volume.
  Note: it is important that every aliquot contains the same sample volume and to use the same kind of reaction vials, since unequal sample volumes may affect the concentration of the measurand due to adsorption.
3. Place aliquots #1–6 at −80°C.
4. Thaw aliquots #2–6 and store again at −80°C.
  Note: thaw for 2 h at room temperature and next store the sample at least 12 h at −80°C for each freeze/thaw cycle.
5. Thaw aliquots #3–6 and store again at −80°C.
6. Thaw aliquots #4–6 and store again at −80°C.
7. Thaw aliquot #5–6 and store again at −80°C.
8. Thaw aliquot #5–6 and store again at −80°C.
9. Thaw aliquot #6 and store again at −80°C.
10. Thaw aliquot #6 and store again at −80°C.
11. At time point 0, store aliquots #7–12 at room temperature and another six aliquots #13–18 at 4°C.
12. At time points *t* = 1 h, *t* = 2 h, *t* = 4 h, *t* = 24 h, *t* = 72 h, *t* = 168 h, transfer one sample stored at each temperature, RT and 4°C, to −80°C.
13. Store aliquot #19 at −20°C during 1 month before transfer to −80°C.
14. Thaw all aliquots for a given sample simultaneously and analyze them in duplicates in the same run.
15. Insert raw data of aliquots #1–19 (replicates of observed concentrations) in the Excel file Data Sheet S4 in Supplementary Material. The file calculates the mean value, SD, and coefficient of variation (%CV) for both the observed concentration and normalized concentration. Note: the SD for the storage conditions and the freeze/thaw aliquots should be within the acceptance criteria for the precision defined in the fit-for-purpose.

The above conditions tested should only serve as an example and the can be modified to better suit the environment and different routine handling of samples at the individual laboratories.

## Internal Quality Control Program

The experiments in a validation are usually performed within a month time and therefore the results represent a kind of snapshot of the performance characteristics of the method. To ascertain that the quality does not degrade over time an internal quality control program should be initiated before the assay is taken into service. The results from quality control samples should be used to determine if a run is accepted and the objective multi rules presented by Westgard should be used (19).

## Summary

In the present study, we present SOPs for validation of assays for biochemical markers together with a template for validation reports. Although this study is part of a project on biomarkers for AD and PD, the SOPs and validation report is generalizable to biomarker assays in any field of clinical medicine. The main focus for the presented SOPs has been on validation parameters relevant to immunochemical methods such as ELISA and related techniques for determination of the concentration of an analyte in a biofluid. Still, many of the parameters are generic and the SOPs could be used outside the realm of immunochemistry. It should also be stressed that the procedures presented here are practical suggestions on how to collect the information needed to demonstrate that the requirements for a method are fulfilled. As such, they could be used also by persons with limited experience in the field of method validation. We believe that validation of biomarker assays before introduction in clinical routine or implementation in clinical trials is essential to get reliable and interpretable results. Information on assay validation is also important in research reports on novel biomarker candidates.

## Conflict of Interest Statement

The authors declare that the research was conducted in the absence of any commercial or financial relationships that could be construed as a potential conflict of interest. The Review Editor Selina Wray declares that, despite being affiliated to the same institution as author Henrik Zetterberg, the review process was handled objectively and no conflict of interest exists.

## Supplementary Material

The Supplementary Material for this article can be found online at http://journal.frontiersin.org/article/10.3389/fneur.2015.00179
